# Management of intrahepatic splenosis:a case report and review of the literature

**DOI:** 10.1186/s12957-018-1419-1

**Published:** 2018-06-28

**Authors:** Zefeng Xuan, Jian Chen, Penghong Song, Yehui Du, Lijun Wang, Dalong Wan, Shusen Zheng

**Affiliations:** 1 Division of Hepatobiliary and Pancreatic Surgery, Department of Surgery, First Affiliated Hospital, School of Medicine, Zhejiang University, 79# Qingchun Road, Hangzhou, 310003 Zhejiang China; 2 Collaborative innovation center for Diagnosis treatment of infectious diseases, 79# Qingchun Road, Hangzhou, 310003 Zhejiang China; 3 Department of Pathology, First Affiliated Hospital, School of Medicine, Zhejiang University, 79# Qingchun Road, Hangzhou, 310003 Zhejiang China

**Keywords:** Liver neoplasm, Intrahepatic splenosis, Splenectomy, Trauma

## Abstract

**Background:**

Splenosis is the heterotopic autotransplantation and implantation of splenic tissue after splenic trauma or splenectomy. Considering that splenosis often occurs in the mesentery, omentum, and peritoneum, intrahepatic splenosis has seldom been reported. We report a rare case of isolated intrahepatic splenosis in a 54-year-old man who presented with a liver mass thought to be hepatocellular carcinoma.

**Case presentation:**

A 54-year-old man was referred to our hospital for further evaluation of a liver lesion. The patient was asymptomatic and had a history of emergent splenectomy after a high-altitude falling accident. Abdominal contrast-enhanced computed tomography revealed a 4.5 × 3.3 cm lesion that was located in segment IV of the left liver lobe. The lesion had an inhomogeneous enhancement during the arterial phase and diminished enhancement during the portal and equilibrium phases. Similar radiological features were also observed on a contrast magnetic resonance imaging scan. Partial hepatectomy was performed with the suspicion of hepatocellular carcinoma. Pathological examination of the liver specimen revealed intrahepatic splenosis.

**Conclusion:**

Splenosis should be considered in differential diagnosis of a liver mass discovered years after splenic trauma or surgery. A proposed scoring system may be helpful in evaluating the suspicious degree of intrahepatic mass to be splenosis. Invasive treatments are not recommended for asymptomatic patients, since the splenosis can provide beneficial immunologic function.

## Background

Splenosis is the heterotopic autotransplantation of splenic tissue throughout the peritoneal and pelvic cavities, even the thoracic cavity, following splenic trauma or elective splenectomy [1]. The splenic fragments usually seed onto exposed vascularized peritoneal surface, receiving blood supply from the surrounding tissue. Intrahepatic splenosis is quite rare, as the majority of splenosis reported in the English literature was found to be located in the mesentery, omentum, and peritoneum [2]. The lack of typical radiological features makes it difficult to distinguish splenosis from liver tumors and reach a correct diagnosis. Herein, we present a case of isolated intrahepatic splenosis and summarize the relevant radiological and pathological characteristics. On the basis of literature review, imaging techniques that may contribute to the diagnosis and appropriate treatment measures are also discussed.

## Case presentation

**Fig. 1:**
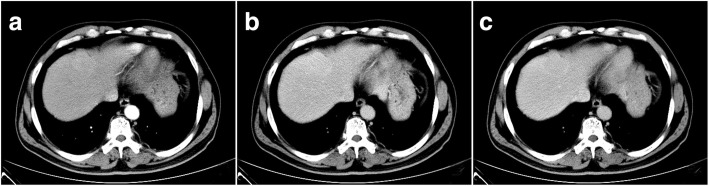
Contrast-enhanced computed tomography scan of intrahepatic splenosis. The lesion had an inhomogeneous enhancement during the arterial phase (**a**) and diminished enhancement during the portal phase (**b**) and equilibrium phase(**c**)

**Fig. 2:**
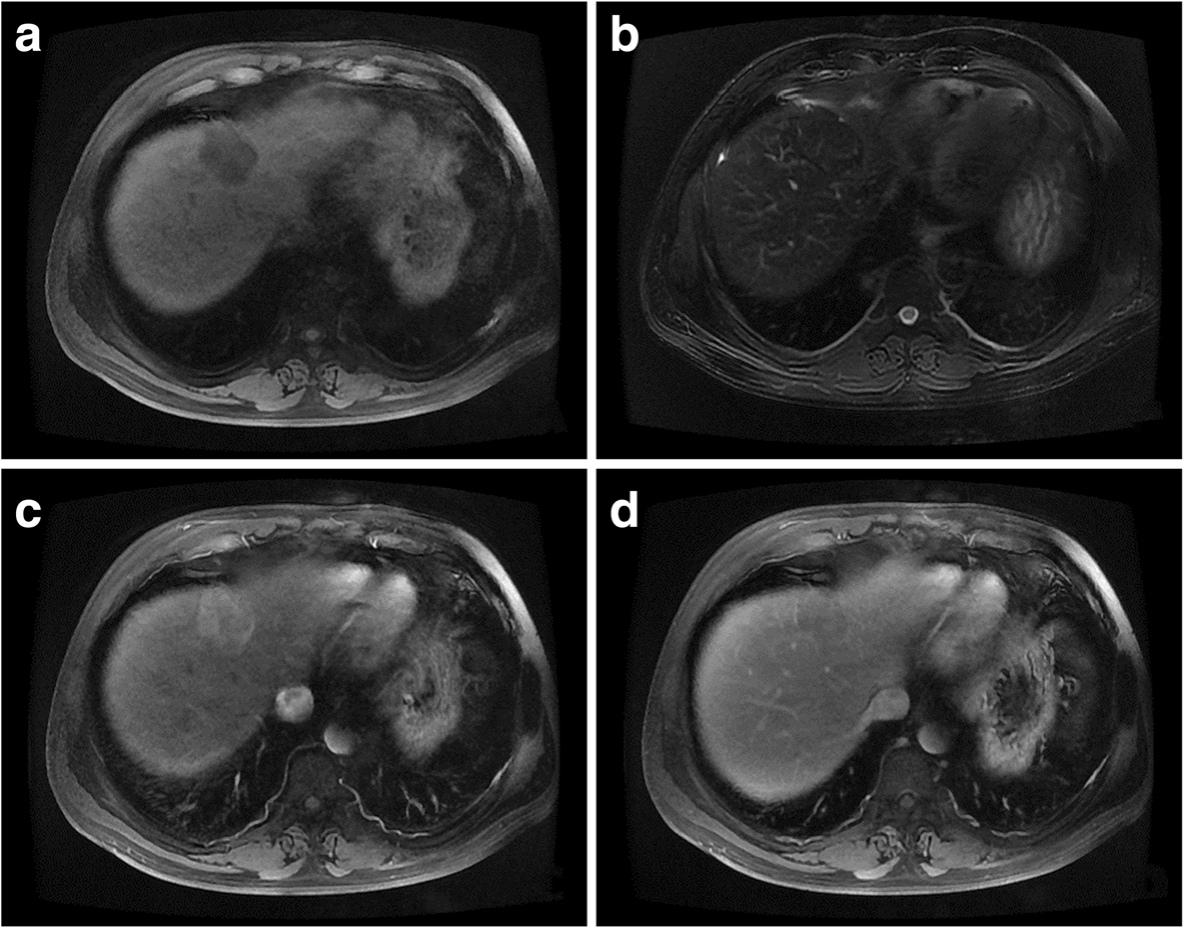
Magnetic resonance imaging scan of intrahepatic splenosis. Note a slightly hypointense mass on both T1-weighted (**a**) and T2-weighted (**b**) images. After the injection of gadoxetic acid, the mass became strongly heterogeneous and hyperintense during the arterial phase (**c**) and relatively hypointense during the portal phase (**d**)

A 54-year-old Chinese male was referred to our hospital for further evaluation of a liver mass, which was discovered incidentally during routine physical examination in a local hospital. The patient had a 10-year history of hypertension and was diagnosed with diabetes mellitus approximately 5 years before. He denied history of liver cirrhosis and hepatitis B virus (HBV) or hepatitis C virus (HCV) infection. The patient underwent splenectomy 5 years earlier owing to a high-altitude falling accident. No mass was identifiable on abdominal palpation exam. Serum tumor markers (alpha-fetoprotein, CA199, and CA125) were within the normal range. Abdominal ultrasonography (US) revealed a 5 cm iso-echoic lesion that located in the left hepatic lobe near the capsule. A 1.2 cm gallstone was also observed. An abdominal plane-computed tomography (CT) scan revealed an oval, slightly hypodense mass located in segment IV of the left liver lobe measuring 4.5 × 3.3 cm. The lesion had an inhomogeneous enhancement during the arterial phase and diminished enhancement during the portal and equilibrium phases on a contrast-enhanced CT scan (Fig. 1). Abdominal magnetic resonance imaging (MRI) showed a slightly hypointense mass on both T1- and T2-weighted images, which appeared slightly hyperintense on diffusion-weighted images. After the injection of gadoxetic acid, the lesion appeared strongly heterogeneous and hyperintense during the arterial phase and relatively hypointense during the portal and equilibrium phases (Fig. 2). An indication of a pseudo-capsule was also observed. Partial hepatectomy and cholecystectomy were performed with the suspicion of hepatocellular carcinoma (HCC).

**Fig. 3:**
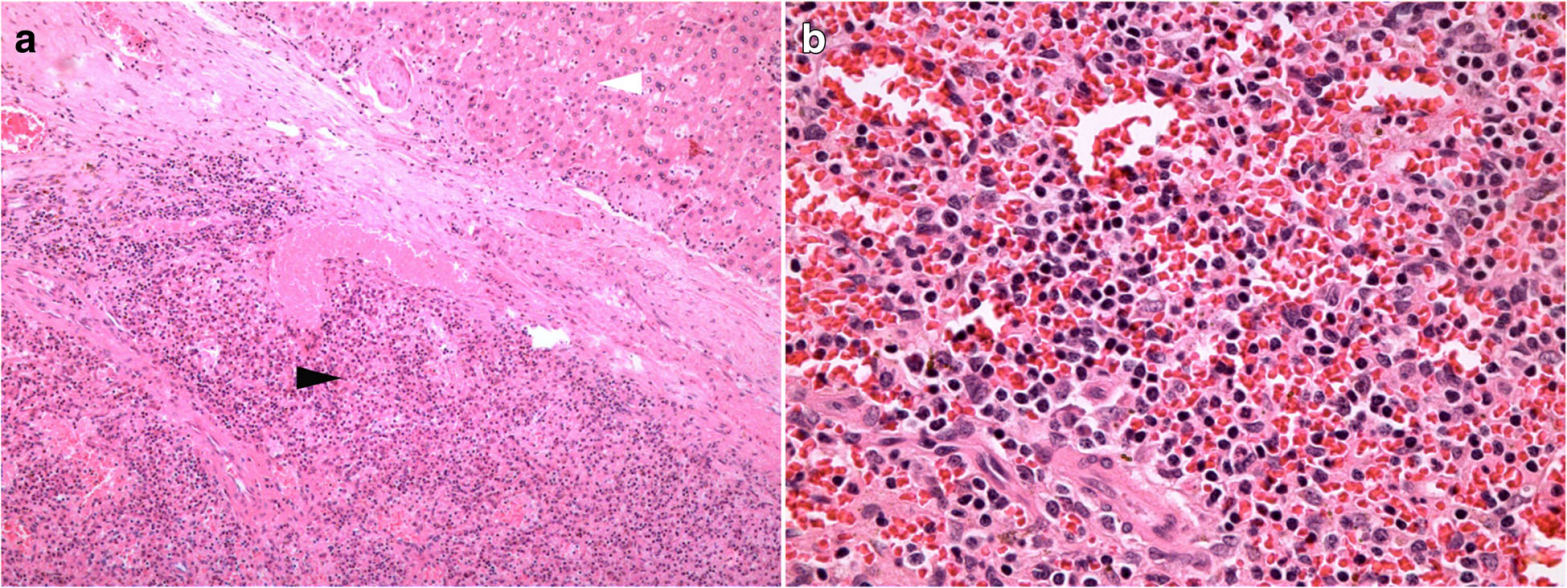
Histopathological features of intrahepatic splenosis. Hematoxylin and eosin staining. **a** A capsule clearly separated the liver (white arrow) and spleen (black arrow) parenchyma, × 100. **b** Intrahepatic splenosis with lymphoid tissue hyperplasia and sinusoidal structures, × 400

During the operation, the intrahepatic mass was found to be located in segment IV of the liver, measuring 4.0 cm in diameter. It was completely embedded in the liver tissue, and no other mass was found. Postoperative hematoxylin and eosin staining revealed sinusoidal structures and lymphoid tissue hyperplasia. A capsule separating the spleen tissue from liver tissue could be clearly detected (Fig. 3), which confirmed intrahepatic splenosis. Detailed immunohistochemical staining showed positivity for CD3 and CD20, specific markers for lymphocyte T cells and B cells, respectively. Meanwhile, the expression of the Ki-67 antigen was quite limited. The polyclonal nature of the lymphocytes and the low proliferation activity further confirmed the benign characteristic of the mass, as malignant tumors are always monoclonal with active proliferation. The patient discharged uneventfully after the operation, and no symptoms of recurrence have been observed during 2 years of follow-up.

## Literature review

We searched the PubMed and Scopus databases for relevant English literature from the year 2000 through March 2018 using the Medical Subject Headings (MeSH) “Hepatocellular Carcinoma,” “Liver Neoplasm,” and “Splenosis.” In total, 37 cases of intrahepatic splenosis were identified and reviewed [3–37]. Characteristics of the cases such as age and sex of patients, clinical symptoms, diagnostic modality, and location of the masses were reviewed and analyzed (Table 1). There were 31 (83.8%) male and 6 (16.2%) female patients, and the mean age of the patients was 49.2 years (ranging from 21 to 73 years).

**Table 1:** Clinical data of 37 cases of intrahepatic splenosis

Author, year	Age (years), gender	Trauma, splenectomy	Liver diseases	Time interval (years)	Symptoms	Diagnostic modality	Number	Subcapsular location	Capsule	Diagnostic hypothesis	Segment, size (mm)	Invasive measure	Follow-up
Teles 2018 [3]	73, M	Splenectomy	No	N/D	Lower back pain	US, CT, MRI scintigraph	2	Yes	Yes	hepatic neoplasia	II, III 36	Surgery	No symptoms for 2 years
Wang 2017 [4]	54, M	Both	HBV	23	Abdominal pain	US, CT, MRI	1	Yes	Yes	HCC	Right posterior lobe 31 × 27	Surgery	No symptoms for 18 months
Wang 2017 [5]	42, M	Both	HBV/BCV	16	Lower back pain	CT, MRI	1	Yes	Yes	HCC	IV 3 × 3	Surgery	N/D
Keck 2017 [6]	66, M	Both	HCV	N/D	No	MRI	2	Yes	N/D	HCC	VII, VIII 53	Biopsy	N/D
Jereb 2016 [7]	22, M	Both	No	18	No	US, CT, MRI	5	Yes	N/D	Liver metastases	II, VI, VII 26 × 26	Surgery	N/D
He 2016 [8]	51, M	Both	No	20	No	US, CT, MRI	2	N/D	N/D	HCC	Left lobe, right lobe 33 × 26	Biopsy	N/D
Liu 2015 [9]	33, M	Both	No	30	No	US, CT, MRI	3	Yes	N/D	HCC/Liver metastases	III, Right lobe 42 × 30	Biopsy	No symptoms for 2 years
Li 2015 [10]	67, F	Both	HCV, cirrhosis	5	No	CT, MRI angiography	1	N/D	Yes	HCC	Left lobe	Surgery	No symptoms for 3 years
Sato 2014 [11]	58, M	Neither	HCV, cirrhosis	No	No	US, CT, MRI	1	Yes	Yes	HCC	Right lateral lobe 39 × 30	Surgery	No symptoms for 1 year
Levi Sandri 2014 [12]	54, M	Both	HBV, cirrhosis	25	N/D	CT	1	N/D	Yes	HCC	III 45 × 35 × 15	Surgery	N/D
Leong 2013 [13]	56, M	Both	No	25	Abdominal pain	US, CT, MRI PET	1	N/D	N/D	Neuroendocrine tumor	III 46 × 37 × 31	Surgery	No symptoms for 6 months
Krawczyk 2013 [14]	39, F	Both	No	N/D	Abdominal pain	CT, MRI, scintigraphy	1	Yes	N/D	Hepatocellular adenoma	II 32 × 20	No	No symptoms for 3 months
Inchingolo 2013 [15]	53, M	Both	Non alcoholic steatohepatitis	33	No	US, CT, MRI	1	Yes	N/D	HCC/hepatic adenoma	III, IV 35	Surgery	N/D
Liu 2012 [16]	49, F	Both	No	20	Subxiphoid pain	US, CT	3	Yes	N/D	Liver tumor	Left lateral lobe 50 × 50	Surgery	No symptoms for 4 months
Liu 2012 [17]	38, M	Both	HBV	14	No	US, CT	1	Yes	Yes	Liver tumor	Left lateral lobe 33 × 27	Surgery	N/D
Kang 2011 [18]	54, M	Both	No	15	No	US, CT, MRI PET-CT	2	Yes	Yes	Liver metastatic nodules	II 23 × 19	Surgery	N/D
Mescoli 2010 [19]	68, F	Neither	Hepatitis, cirrhosis	No	Abdominal pain	US, CT, MRI	3	N/D	N/D	Liver tumor	III, V, VII 150	Biopsy	N/D
Mescoli 2010 [19]	54, M	Splenectomy	No	12	No	CT, PET-CT	1	N/D	No	Liver metastatic nodule	Left lobe 30	Surgery	No symptoms for 8 months
Yu 2009 [20]	54, M	Both	No	20	No	US, CT, MRI	1	Yes	Yes	N/D	Left lobe 40	Surgery	No symptoms for 6 months
Menth 2009 [21]	43, M	Both	HCV, cirrhosis	30	Fatigue	US, CT, MRI angiography, scintigraphy	Multiple	Yes	N/D	HCC	II 36	Biopsy	No symptoms for 9 months
Kashgari 2009 [22]	52, M	Both	HCV, cirrhosis	30	No	US, MRI	1	Yes	N/D	HCC	VII 21 × 15	Biopsy	No symptoms for 4 months
Abu Hilal 2009 [23]	60, M	Both	HCV, cirrhosis	46	Flu-like symptoms	US, CT, MRI	1	Yes	N/D	HCC	VII 30	Surgery	No symptoms for 2 years
Yeh 2008 [24]	64, M	Both	HCV	8	No	US, CT, MRI angiography	1	Yes	Yes	HCC	VI 25	Surgery	N/D
Nakajima 2008 [25]	41, M	Both	N/D	21	Abdominal pain and diarrhea	US, CT, MRI	1	Yes	N/D	N/D	VI N/D	Biopsy	N/D
Imbriaco 2008 [26]	39, M	Both	No	24	Abdominal pain	US, CT, MRI	Multiple	Yes	N/D	Liver metastatic nodules	Left lobe, right lobe 30	Surgery	N/D
Grande 2008 [27]	41, M	Both	No	35	No	US, CT, scintigraphy	Multiple	Yes	N/D	N/D	VII 45	No	N/D
Choi 2008 [28]	32, M	Both	HBV	26	No	CT, MRI angiography	3	Yes	Yes	HCC	IVa, IVb, VI 30	Surgery	N/D
Brancatelli 2005 [29]	38, F	Both	No	32	No	CT, MRI scintigraphy	1	Yes	N/D	Liver adenoma	Left lobe 50	Biopsy	N/D
Zhao 2004 [30]	49, M	Both	No	17	No	US, CT	1	Yes	Yes	Liver adenoma/HCC	VII 50 × 30 × 30	Surgery	No symptoms for 1 year
Kondo 2004 [31]	55, M	Both	HCV	31	No	US, CT, MRI(SPIO) angiography	2	N/D	N/D	HCC	VII 35 × 35	Biopsy	N/D
Izzo 2004 [32]	60, M	Both	HCV	43	Jaundice	US, CT, MRI	1	N/D	N/D	HCC	Near the hilum 60	Biopsy	N/D
Di Costanzo 2004 [33]	58, M	Both	HBV, cirrhosis	46	No	US, CT, scintigraphy	1	Yes	N/D	HCC	III 48	Biopsy	N/D
Di Costanzo 2004 [33]	48, F	Both	HCV, cirrhosis	41	No	US, CT	1	Yes	Yes	HCC	III 31	Biopsy	N/D
Kim 2003 [34]	43, M	Both	HBV, cirrhosis	21	No	US, CT, angiography	1	Yes	Yes	HCC	Right lobe 30	Surgery	N/D
Pekkafali 2002 [35]	21, M	Both	No	15	Epigastric pain	US, CT, MRI scintigraphy	1	Yes	Yes	No	Left lobe 34 × 23	No	N/D
Lee 2002 [36]	43, M	Both	HBV, cirrhosis	20	Fatigue	US, CT, angiography	1	Yes	Yes	HCC	VI 33 × 20	Surgery	N/D
De Vuysere 2000 [37]	50, M	Both	No	34	No	US, CT, MRI(SPIO)	3	N/D	Yes	No	Left lobe, right lobe 60	Biopsy	N/D

*HBV* hepatitis B virus, *HCV* hepatitis C virus, *HCC* hepatocellular carcinoma, *US* ultrasonography, *CT* computed tomography, *MRI* magnetic resonance imaging, *PET* positron emission tomography, *M* male, *F* female, *N/D* not disclosed

^a^Time interval: time elapsing between trauma/splenectomy and diagnosis of intrahepatic splenosis

^b^When multiple lesions, only the size of the largest one was presented

^c^Surgery: included laparoscopic resection and laparotomy

In the 37 documented cases, 35 (94.6%) patients had histories of trauma or/and splenectomy, and the mean time elapsing between trauma/splenectomy and diagnosis of intrahepatic splenosis was 24.9 years (ranging from 5 to 46 years). A total of 20 (54.1%) patients had related liver diseases, among which 8 (40%) had HBV infection, 11 (55%) had HCV infection, and 11 (55%) had cirrhosis. Most of the patients were asymptomatic upon admission, except for 6 (16.2%) who had abdominal pain. US, CT, and MRI were common imaging techniques, but they did not clearly differentiate intrahepatic splenosis from other liver lesions, such as HCC, liver metastases, or liver adenoma. Scintigraphy was used in 7 (18.9%) patients, and in 3 (42.9%) of them, the imaging led to the correct diagnosis without further invasive measures. The majority of intrahepatic splenosis were located in the subcapsular region of the liver, surrounded by capsules. A total of 34 (91.9%) patients had undergone invasive procedures. Surgery in 21 (61.8%) patients, including laparoscopic resection and laparotomy, was the most common invasive procedure followed by biopsy in 13 (38.2%).

## Discussion

Splenosis represents the heterotopic autotransplantation and implantation of splenic tissue after elective splenectomy or traumatic spleen rupture. Once considered to be a rare condition, a recent estimated incidence is up to 67% of patients who have a history of splenic rupture or surgery [38]. Intrahepatic splenosis is still rare, as most of the splenoses were located in the mesentery, omentum, or peritoneum. Except for some extraordinary cases, almost all of the cases with intrahepatic splenosis have a history of splenic trauma or splenectomy [11, 19]. Hence, intrahepatic splenosis should be taken into consideration in patients with a relevant history, especially if the mass is found to be located close to the liver capsule.

The absence of typical radiological features makes it difficult to reach a correct diagnose with common imaging techniques, such as US, CT, and MRI. As a result, intrahepatic splenosis can be confused with HCC, adenoma, or other liver diseases, leading to unnecessary surgery or other invasive treatments. Therefore, more sensitive novel methods to diagnose intrahepatic splenosis are needed. Scintigraphy with sensitive technetium-99 m-labeled heat-denatured red blood cells (Tc-99 m-DRBC) is reported to be the most specific and efficient diagnostic method [20]. As approximately 90% of damaged erythrocytes will be trapped by splenic tissue, remarkable differences in uptake of the radioactive isotope can be observed between intrahepatic splenic tissue and normal liver tissue. Krawczyk et al. [14], Grande et al. [27], and Pekkafali et al. [35] reported three cases that successfully avoided invasive treatments by using Tc-99 m-DRBC scintigraphy. Scintigraphy with sulfur colloid is considered to be another useful diagnostic method, but has a lower sensitivity in identification of splenosis [39]. Superparamagnetic iron oxide (SPIO) contrast magnetic resonance imaging may be helpful for the diagnosis of splenosis. As reported, intrahepatic splenosis will remain hyperintense relative to the liver parenchyma, while HCC will become hypointense after the SPIO administration [37].

In fact, most of the cases with intrahepatic splenosis that had been reported were treated with invasive procedures, including biopsy and surgical resection. However, intrahepatic splenosis may be beneficial in the patients who have undergone splenectomy, since it can replace part of the immunologic function of the removed spleen [40]. Hence, conservative treatment is strongly recommended for asymptomatic intrahepatic splenosis, except for some special situations, such as idiopathic thrombocytopenic purpura and Felty syndrome.

In order to avoid unnecessary invasive treatment, accurate diagnosis is essential. Although some novel imaging methods, such as scintigraphy, have shown promising application prospects in diagnosis of intrahepatic splenosis, they will not likely be used worldwide for quite some time. Instead, we think it may be helpful to use a scoring system to evaluate the suspicious degree of intrahepatic mass to be splenosis (Table 2). Compared with the CT/MRI Li-Rads v2017 [41], our scoring system seems to be more effective in diagnosing intrahepatic splenosis. The major imaging features (washout, enhancing “capsule” and threshold growth) of Li-Rads were not enough to distinguish intrahepatic splenosis from liver neoplasm. According to the table, the higher the total score is, the stronger is the possibility that the mass will be splenosis. When the total score is greater than 3, it is better to use biopsy to clarify the diagnosis, instead of taking more aggressive measures directly.

**Table 2:** Suspicious degree of intrahepatic mass to be splenosis

Parameters	Score		Methods
	0	1	
Alpha-fetoprotein	> 400 μg/L for 4 weeks > 200 μg/L for 8 weeks	No	ELISA
Cirrhosis	Yes	No	US, CT, MRI
Hepatitis	Yes	No	ELISA, PCR
Splenic trauma	No	Yes	History taking/ US, CT, MRI
Splenectomy	No	Yes	History taking/ US, CT, MRI
Mass location	Non-subcapsular	subcapsular	US, CT, MRI
Mass capsule	No	Yes	US, CT, MRI
Howell-Jolly and Heinz bodies after splenectomy	Yes	No	Hematological examination

*ELISA* enzyme-linked immunosorbent assay, *US* ultrasonography, *CT* computed tomography, *MRI* magnetic resonance imaging, *PCR* polymerase chain reaction

^a^For alpha-fetoprotein, exclude pregnancy, acute severe hepatitis, embryonic gonad tumors, and other digestive system tumor

## Conclusion

Although isolated intrahepatic splenosis is rarely encountered, it should be taken into account in the differential diagnosis of a liver lesion, especially if the patient has a history of splenic trauma or splenectomy. The proposed scoring system may be useful in diagnosing intrahepatic splenosis when effective diagnostic methods, like scintigraphy and SPIO MRI, are lacking. If intrahepatic splenosis has been confirmed, conservative treatment is strongly recommended for the patient without any symptoms.

## Abbreviations

CT: Computed tomography
ELISA: Enzyme-linked immunosorbent assay
PCR: Polymerase chain reaction
F: Female
HBV: Hepatitis B virus
HCC: Hepatocellular carcinoma
HCV: Hepatitis C virus
M: Male
MeSH: Medical Subject Headings
MRI: Magnetic resonance imaging
N/D: Not disclosed
PET: Positron emission tomography
SPIO: Superparamagnetic iron oxide
Tc-99 m-DRBC: Technetium-99 m-labeled heat-denatured red blood cells
US: Ultrasonography
